# Differential Sensitivity of Regulatory and Effector T Cells to Cell Death: A Prerequisite for Transplant Tolerance

**DOI:** 10.3389/fimmu.2015.00242

**Published:** 2015-05-19

**Authors:** Sylvaine You

**Affiliations:** ^1^Université Paris Descartes, Sorbonne Paris Cité, Paris, France; ^2^INSERM U1151, Institut Necker-Enfants Malades, Paris, France; ^3^CNRS UMR 8253, Institut Necker-Enfants Malades, Paris, France

**Keywords:** immunotherapy, tolerance, monoclonal antibodies, depletion, Foxp3^+^ Tregs, resistance, CD3

## Abstract

Despite significant progress achieved in transplantation, immunosuppressive therapies currently used to prevent graft rejection are still endowed with severe side effects impairing their efficiency over the long term. Thus, the development of graft-specific, non-toxic innovative therapeutic strategies has become a major challenge, the goal being to selectively target alloreactive effector T cells while sparing CD4^+^Foxp3^+^ regulatory T cells (Tregs) to promote operational tolerance. Various approaches, notably the one based on monoclonal antibodies or fusion proteins directed against the TCR/CD3 complex, TCR coreceptors, or costimulatory molecules, have been proposed to reduce the alloreactive T cell pool, which is an essential prerequisite to create a therapeutic window allowing Tregs to induce and maintain allograft tolerance. In this mini review, we focus on the differential sensitivity of Tregs and effector T cells to the depleting and inhibitory effect of these immunotherapies, with a particular emphasis on CD3-specific antibodies that beyond their immunosuppressive effect, also express potent tolerogenic capacities.

## Introduction

The clinical success of transplantation depends on a life-long use of immunosuppressive drugs that depress the immune system in a global manner. These non-specific therapies significantly reduced the incidence of acute rejection but the benefit in terms of chronic rejection or long-term graft survival is uncertain. Indeed, the induction of a permanent immunosuppressive state, which may be excessive in some patients, is associated with significant toxicity and morbidity with increased incidence of cancer, opportunistic infections, and cardiovascular diseases. Therefore, alternative approaches have been developed with the objective of targeting the unwanted immune responses to the transplanted organ while sparing the beneficial functions of the immune system, i.e., inducing operational tolerance. Strategies include the use of monoclonal antibodies (Abs) or fusion proteins that block T cell activation, the infusion of donor antigens (DST), the induction of hematopoietic chimerism (bone marrow transplantation), the use of immunomodulatory cytokines or cell therapy (dendritic cells, regulatory T cells). These approaches utilize various mechanisms of peripheral tolerance such as deletion, activation-induced cell death (AICD, apoptosis), anergy, immune deviation, and/or induction of regulatory T cells (Tregs).

When looking at the data more closely, notably the one gained from studies using monoclonal antibodies or fusion proteins, we can observe that most treatments differentially affect T cell populations. In particular, activated effector T cells and CD4^+^Foxp3^+^ Tregs react differently to the inhibitory or depleting effect of these biological agents. This is of importance as induction of transplant tolerance across full MHC histocompatibility barriers is thought to require the presence of Foxp3^+^ Tregs as well as the physical elimination of effector T cells. In this mini review, we will discuss the differential sensitivity of T cell subsets to immunointervention with a particular focus on CD3 antibody-based therapy in transplantation.

## Differential Sensitivity of Effector and Regulatory T Cells to Immunotherapy

Alloreactive T cells are present in naive recipients and are thus able to recognize alloantigens and mount efficient immune responses against the transplanted organ (1). Therefore, depletion or inhibition of these alloreactive T cells is necessary to promote graft survival (2). However, there is compelling data derived from experimental models suggesting that this step is not sufficient *per se* to induce peripheral tolerance and must be implemented with mechanisms that maintain effective Treg function to control both remaining alloreactive T cells and new thymic emigrants (3). Therapeutic approaches should combine these two capacities to induce permanent allograft acceptance and antigen-specific tolerance.

In most experimental models, the success of therapeutic strategies is associated with the fact they target effector T cells while preserving CD4^+^Foxp3^+^ Tregs from deletion or functional inhibition (Table 1). Indeed, reports both in mice and humans showed that Foxp3^+^ Tregs are relatively spared from the lymphodepleting effect of anti-thymocyte globulin (ATG) (4–11). ATG treatment increased the frequency and the functional activity of Tregs and, in some models, *de novo* generation of antigen-specific Tregs has been demonstrated (12). Similarly, Treg number and activity were not significantly affected by treatment with anti-lymphocyte serum (ALS) (13).

**Table 1:** **Treg resistance to inhibition/depletion mediated by biological agents in transplantation**.

Strategies	Transplanted organs	Reference
ATG	Skin	(4, 5, 8–11)
	Kidney	
	GVHD	
	Pancreatic islets	
ALS	Skin	(14)
Non-depleting CD4 Abs	Skin	(15–18)
	Heart	
CD40L Abs	Skin	(16, 19–22)
	Pancreatic islets	
	GVHD	
CD28 Fab′fragment	Heart	(23–25)
	Kidney	
Agonist IL-2/Fc + antagonist IL-15/Fc	Heart	(26)
CD3 Abs	Pancreatic islets	(27–29)
	Heart	

In the same vein, short course of non-depleting CD4 and CD8 Abs (combined with CD40L Abs in some MHC-mismatched conditions) efficiently promoted tolerance by inhibiting alloreative T cells while promoting regulatory mechanisms (30). The use of transgenic models revealed that non-depleting CD4 Abs leave intact the proliferation of allospecific Tregs while abrogating the expansion of allospecific effector T cells *in vitro* and *in vivo* (15, 16). Natural and induced CD4^+^Foxp3^+^ Tregs play crucial role in inducing and maintaining transplant tolerance through mechanisms of “linked suppression” and “infectious tolerance” (17, 30–34). This occurs when a Foxp3^+^ Treg and a Foxp3^−^ conventional T cell recognize their respective alloantigens presented by the same APC, thereby inducing a tolerizing signal within the conventional T cells which acquire regulatory properties instead of differentiating into alloreactive effector T cells. These “secondary” Tregs can propagate the tolerance state by inducing “tertiary” Tregs. The infectious nature of the process and the critical role of CD4^+^Foxp3^+^ Tregs have been confirmed in a B6.Foxp3^hCD2^ transgenic mouse model (18). Importantly, the continuous presence of the alloantigens is required to ensure dominance by Tregs, which accumulate in tolerated grafts where they efficiently control alloreactive effectors (18, 35). Immunoregulatory cytokines also contribute to CD4/CD8 Ab-induced tolerance, notably TGF-β, which has been detected in tolerated grafts and can promote the *de novo* generation of Foxp3^+^ Tregs (35–37).

Costimulatory blockade is another well-established strategy that targets mature peripheral T cells and manipulates the immune system in a manner that favors Treg development and abrogates alloreactive responses (16, 19–21). It has been reproducibly shown that administration of the fusion protein CTLA-4Ig and/or antibodies to CD40L (CD154), BTLA, ICOS, OX-40, or CD28 efficiently inhibits allogeneic effector T cell activation and expansion (38). Combined with each other, with DST or immunosuppressive drugs such as rapamycin, these biological agents induce long-term acceptance of allogeneic organs and tissues in mice and non-human primates (23, 24, 39–43). Interestingly, besides promoting T cell unresponsiveness, some of these agents also induce T cell apoptosis, which is partial and transient yet mandatory for tolerance induction (40, 44–47). This is well illustrated by the fact that recipient mice transgenic for the anti-apoptotic molecule Bcl-x_L_ are refractory to the therapeutic effect of CTLA-4Ig and CD40L Abs and thereby reject skin or heart allografts (40, 44). Importantly, several reports show that alloreactive effector T cells are the primary targets of these therapeutic agents. For instance, in a major MHC-mismatched skin graft model, combination of CD40L Ab to DST promoted transplant tolerance through the selective depletion of alloantigen-specific CD8^+^ T cells (47). Another study revealed that CD40L Ab can fix complement through its Fc fragment and mediates selective depletion of activated T cells, which, in association with rapamycin, induce long-term graft survival (46). The use of CD40L F(ab′)_2_ fragments or C3- and Fc receptor-deficient recipients completely abrogated the therapeutic effect. Clonal deletion was illustrated by the disappearance of effector T cells expressing specific TCR Vβ families after treatment with CD40L Ab, CTLA-4Ig, and rapamycin (48).

Regulatory T cells resist the inhibitory/depleting effect of costimulation blockade and they play a crucial role in the development and the maintenance of transplant tolerance (38). Indeed, their deletion abrogates tolerance and leads to graft rejection (22, 49). Expansion of thymus-derived Tregs as well as *de novo* generation of Tregs have been reported and infectious tolerance has been proposed as a key mechanisms allowing long-term allograft acceptance (30, 50). This positive effect on Tregs is particularly observed when rapamycin is combined to costimulatory blockade, which is not the case when calcineurin inhibitors (CNIs: cyclosporine A, tacrolimus) are used (20, 48, 51). It has been suggested that CNIs have a detrimental effect on CD4^+^Foxp3^+^ Treg homeostasis as they block NFAT activation and IL-2 production in contrast to rapamycin which promotes Treg function and expansion (52–55). Using endoscopic confocal microscopy and color-coded T cell subsets, Fan et al. showed that the number of Tregs detected within the allografts and peripheral blood of mice treated with CD40L Abs and rapamycin was similar to that of untreated recipients but the number of effector T cells was drastically reduced after treatment (20). In a graft-versus-host disease (GVHD) model, CTLA-4Ig/CD40L Ab treatment prevented the expansion of host-reactive donor T cells but promoted proliferation of Foxp3^+^ Tregs (22). Similar features were observed *in vitro* (21). In the same vein, recent studies in non-human primates showed that treatment with selective CD28 antagonists did not reduce Foxp3^+^ Treg number after transplantation and favored their accumulation within the graft (23, 24). Interestingly, selective CD28 blockade prevented formation of the immune synapse between alloreative effector T cells and APC by inhibiting the TCR Stop signal while it increased Treg contact time with APC and induced calcium mobilization which translated into enhanced Treg suppressive activity (25). Such effects were not reported for CD80/86 antagonists. As CD28-B7 interaction is mandatory for Treg development and homeostasis, these results together with the data from the phase III BENEFIT study, reporting an increased occurrence of acute graft rejection episode and lymphoproliferative disorders in renal transplant patients treated with belatacept, have raised concerns about the negative impact of CD28-B7 blockade on regulatory mechanisms and graft survival (56–58).

Preservation of Tregs homeostasis and function has been demonstrated in other tolerance-promoting protocols. Tregs were resistant to the lytic effect of a regimen combining rapamycin, agonist IL-2/Fc, and antagonist mutant IL-15/Fc fusion proteins that depleted activated effector T cells through apoptosis and antibody-dependent pathway and promoted long-term engraftment of allogeneic skins and pancreatic islets (26). However, application of this treatment in non-human primates only permitted a modest prolongation of cardiac allograft survival and did not reduce the pool of effector or memory CD8^+^ T cells although Tregs were found in increased numbers in the blood of treated animals (59).

## A Distinct Effect of CD3-Specific Antibodies on Tregs and Effector T Cells

CD3-specific monoclonal Abs act through distinct mechanisms that are not mutually exclusive (60). The antibodies transiently deplete T cells although they display no or little complement-dependent and antibody-dependent cellular cytotoxicity. Mechanism of redirected cell lysis due to the ability to crosslink CD3 molecules expressed by two different cells (cytotoxic CD8^+^ T cells on one side and other target T cells on the other side) has been demonstrated (61). However, T cell depletion mostly results from AICD. Indeed, through its F(ab′)_2_ portion, CD3 Ab can not only induce antigenic modulation (i.e., internalization or shedding of the TCR/CD3 complex) but also can transduce signals into T cells, which activate them and promotes their apoptosis (AICD) or anergy. Importantly, non-FcR binding human or mouse CD3 Abs are not passive blocker of TCRs, they can deliver partial TCR signaling and retain their full therapeutic properties without the toxicity associated with the parental CD3 Ab (62–65). This has been the rationale of using humanized CD3 antibodies in the clinic in type 1 diabetes patients (66–68).

We and other accumulated evidence showing that CD3 Abs preferentially target and deplete activated effector T cells while preserving CD4^+^Foxp3^+^ Tregs. This finding was initially suggested by indirect observations in autoimmunity showing that CD3 Abs exert their tolerogenic capacities only when applied at the time of ongoing autoreactive responses or established disease (60, 62, 69). These data highlighted the importance of the immune activation status toward the relevant antigen(s) at the time of treatment. We obtained the same results when translating CD3 Ab therapy to the transplantation field. In experimental models of fully mismatched pancreatic islets or cardiac allografts, low-dose treatment of recipient mice at the time of transplantation prolonged graft survival but rejection occurred systematically (27, 28). By contrast, when postponing CD3 Ab administration after transplantation, in a defined therapeutic window, long-term survival and immune tolerance were observed. This therapeutic window corresponded to the time of effector T cell priming to the alloantigens characterized by the occurrence of anti-donor T cell responses and infiltration of allografts by significant number of alloreative T cells (27, 28).

Several experimental data argue for the preferential elimination of activated effector T cells by CD3 antibodies. First, using a transfer model where OVA-specific OT-I CD8^+^ T cells were primed *in vivo* by SIINFEKL(OVA)-loaded dendritic cells, we showed that only highly dividing OT-I cells entered apoptosis after CD3 Ab administration applied 6 days after cell infusion. Resting endogenous CD8^+^ T cells were not affected by the treatment (27). Second, in a murine model of GVHD, injection of CD3 F(ab′)_2_ fragments selectively depleted activated donor T cells that underwent cell division upon recipient antigens recognition (70). Third, using another non-Fc receptor binding CD3 Ab (145-2C11-IgG3), Penaranda et al. showed that adoptively transferred effector Th1 cells were much more sensitive to the depleting effect of CD3 Abs than endogenous naïve T cells (71). Fourth, *in vitro* experiments also showed that non-Fc receptor binding humanized CD3 Abs displayed an increased ability to deplete activated human T cells through AICD (63, 72).

In contrast to effector T cells, Foxp3^+^ Tregs appear resistant to CD3 Ab-mediated cell death. Indeed, in terms of absolute number, the decrease in Foxp3^+^ T cells is always much less pronounced than that observed for conventional Foxp3^−^ T cells and, in some situations, is not altered at all (27, 71, 73, 74). Consequently, the frequency of Tregs significantly increases in peripheral blood and secondary lymphoid organs after CD3 Ab treatment. Similar results were reported for CD4^+^CD25^+^CD62L^+^ T cells (75). In most reports, no evidence of conversion of conventional T cells into Tregs as well as significant expansion of natural Tregs were reported except in a recent article by Valle et al. showing an increase in Treg count in the blood of treated mice resulting from *in vivo* proliferation (73). The reduced sensitivity of CD4^+^Foxp3^+^ Tregs to CD3 Abs was illustrated at the molecular level by a transcriptome analysis showing that gene expression profile (related to the TCR signaling pathway) of Tregs was much less impacted than the one of CD4^+^Foxp3^−^ T cells after *in vivo* treatment with CD3 Abs (73). All these data concur to show that Tregs resist CD3 Ab-induced AICD.

This feature of Tregs is crucial for CD3 Ab-induced transplant tolerance. Both in the pancreatic islet and the cardiac allograft models, we observed that the proportion of CD4^+^ and CD8^+^ T cells significantly decreased at the periphery and within the grafted organs after administration of CD3 Ab F(ab′)_2_ fragments (27, 28, 76). However, depletion of Foxp3^+^ Tregs was very limited as compared to that of CD8^+^ T cells or CD4^+^Foxp3^−^ T cells (27, 28). Consequently, peripheral and intragraft Treg proportion increased after CD3 Ab therapy (27–29, 76). Foxp3^+^ Tregs isolated from CD3 Ab-treated tolerant mice and adoptively transferred into a RAG^−/−^ recipients were able to prevent graft rejection mediated by naïve spleen cells toward islet allografts from the same donor but not from a third-party donor, thereby indicating that these Tregs play a key role in sustained antigen-specific transplant tolerance over long term (27).

## Mechanisms of Resistance

All these data concur to show that Foxp3^+^ Tregs are less sensitive than effector T cells to the apoptotic or inhibitory effect of therapeutic antibodies. The mechanisms underlying this resistance are not completely elucidated. In the context of ALS treatment, it has been proposed that this resistance to cell death is dependent on the costimulatory molecule OX-40 as well as the anti-apoptotic molecule Bcl-x_L_ that was detected at higher levels in Tregs than in conventional T cells (13, 14). Another mechanism that may contribute to Treg resistance to apoptosis is related to studies suggesting that they express lower levels of FasLigand (CD95L) upon stimulation as compared to conventional Foxp3^−^ T cells (77). Abrogation of Foxp3 expression (in Scurfy mice) rescues FasL expression at the level comparable to those of conventional T cells, suggesting that Foxp3 controls, at least in part, the expression of FasL and AICD. Accordingly, naïve Tregs has been shown to be more resistant to Fas-induced cell death than conventional T cells (78, 79).

In the context of CD3 antibody therapy, the differential effect of the antibody on effector versus regulatory T cells appears paradoxical as the CD3 molecular complex is expressed on all T cells. The reasons explaining this differential effect are still not elucidated. However, recent studies suggested that both human and mouse Tregs harbor less CD3 molecules (comprising the CD3ε chain, which is that target of the therapeutic antibodies) on their surface than do CD4^+^Foxp3^−^ T cells and that this variable expression level correlated with different susceptibility to CD3 Ab-mediated cell death (73, 80). In addition, it has been suggested that Tregs (CD4^+^CD25^+^) possess distinct isoforms of the CD3epsilon chain characterized by an undegraded N-terminal sequence of negatively charged amino acid residues that is associated with a higher activation threshold (80). A diminished expression of signaling molecules downstream of the TCR/CD3 complex has also been reported in Tregs (CD3ζ, ZAP-70, LcK, Vav, and PI3K p110α) as well as a reduced signaling after TCR engagement in response to CD3 antibodies (phospho-ZAP-70, -Akt, -Erk1/2, -PLCγ1) as compared to conventional T cells (CD4^+^CD25^−^) (80, 81). Lastly, the fact that Foxp3^+^ Tregs may express lower levels of FasL upon stimulation than Foxp3^−^ T cells may account for their resistance to the depleting effect of CD3 antibodies that is at least partially mediated by the Fas/FasL pathway (60, 77).

## Concluding Remarks

Several potentially important biological agents have been used to prevent transplant rejection with the aim of inducing transplant tolerance. The intrinsic resistance of Tregs to the depleting effect of these therapeutic tools, which preferentially target effector alloreative T cells, encourages clinical translation of these strategies. Unfortunately, so far, these tolerance-promoting protocols are often disappointing when applied to human transplantation (82). To move faster and to achieve successful translation to the clinical arena in a not too distant future, clinicians and researchers need to build on the drugs currently in clinical development to design new tolerogenic protocols. The challenge is to use these biological tools in a defined therapeutic window to reveal their tolerogenic capacities (i.e., deleting effector/memory alloreactive T cells while harnessing Tregs) in order to minimize/withdraw immunosuppressive drugs without increased risks of acute rejection but in a manner that positively impact on long-term graft survival through the induction of operational tolerance. In that situation, CD3-specific Abs may represent one promising approach as they afford long-term therapeutic effect following a short-term and low-dose administration. Finally, these features are further emphasized by the growing interest on Treg cell therapy in transplantation (83, 84). Indeed, the use of *in vitro* expanded Tregs requires the selection of therapeutic agents that enhance Treg action to enable efficacy of the combined therapy and application to clinical transplantation (85, 86).

## Conflict of Interest Statement

The author declares that the research was conducted in the absence of any commercial or financial relationships that could be construed as a potential conflict of interest.
